# Assessing attentional bias to emotions in adolescent offenders and nonoffenders

**DOI:** 10.3389/fpsyg.2023.1192114

**Published:** 2023-11-24

**Authors:** Mariana Pino, Victor Pardo, Ronald Ruiz, Gabriel González, Mario Alfredo Parra

**Affiliations:** ^1^Psychology Program, Universidad Autónoma del Caribe, Barranquilla, Atlántico, Colombia; ^2^Psychology Program, Universidad de San Buenaventura, Cartagena, Cartagena, Colombia; ^3^Fundación Hogares Claret, Barranquilla, Atlántico, Colombia; ^4^Department of Psychological Sciences and Health, University of Strathclyde, Glasgow, Scotland, United Kingdom

**Keywords:** offenders, adolescence, delinquency, attentional bias, emotional processing

## Abstract

Emotional processing is a cognitive function essential for the interaction of humans with their environment and the development of adaptive behaviors. Adolescent offenders (AOs) express difficulty in cognitive processes linked to emotional processing, which is a response consistently observed during the endogenous (i.e., controlled) control of attention. Less remains understood of the extent to which such atypical responses extend beyond controlled attention and influence exogenous mechanisms (i.e., automatic). This study explores this hypothesis using the recently devised emotional Flanker paradigm. It recruited a group of 39 male AOs and 39 nonoffenders from Barranquilla, Colombia. Assessment consists of an emotional Flanker paradigm administered along with traditional neurocognitive and social cognition tasks. The AOs displayed the well-known attentional bias to threat and a relatively atypical response to emotional targets in which they detected emotions, particularly negative ones, faster than did nonoffenders. Frontal lobe functions account for these effects but not sociodemographic variables nor general cognitive abilities. The results are interpreted in light of evidence suggesting that youngsters with high levels of antisocial behaviors (e.g., callous–unemotional traits) present an enhanced orientation toward distressing stimuli, which is explained by lifelong experiences (e.g., histories of abuse). The findings suggest that environmental influences seemingly exist in the development of these traits, but additional research is required to elucidate the role of cognitive and environmental factors in the development of antisocial behavior.

## 1 Introduction

The escalating situation of Adolescents offenders (Aos) in Colombia is a phenomenon that is historically associated with factors, such as inequality and psychosocial vulnerability, unsatisfied basic needs, or poverty and exclusion from the educational system (Instituto Colombiano de Bienestar Familiar-ICBF, 2012, 2015). Unfortunately, in many cases, these factors include the effects of internal irregular war and postarmed conflict scenarios in geographical areas in which they continue to subsist through illicit economies such as coca cultivation or illegal mining (Ríos et al., 2019). This population is mainly composed of adolescent boys from lower socioeconomic backgrounds who needed to abandon academic studies and, in certain cases, relocate to avoid being recruited by illegal armed groups (Comisión de la Verdad Colombia, 2019; Ríos et al., 2019). In addition, psychosocial background profiles demonstrated that the majority have a life history marked by domestic violence; neglect; cultural, ethnic, or social class discrimination, and abusive use of psychoactive substances (Instituto Colombiano de Bienestar Familiar-ICBF, 2012, 2015; Centro nacional de memoria histórica.: ¡Basta Ya! Colombia: Memoria de Guerra y Dignidad, 2013; Comisión de la Verdad Colombia, 2019; Ríos et al., 2019). Among the most common reasons for their detainment in reeducation and resocialization centers are robbery, homicide, attempted homicide, personal injury, trafficking, manufacture or transport of narcotics, and extortion (Instituto Colombiano de Bienestar Familiar-ICBF, 2012, 2015). In this context of social violence along with the interest of neuroscience in the research on the neurobiological components and processes of social cognition, the scientific study of emotions has proliferated in Colombia. For example, Trujillo et al. (Papp et al., 2016) and Tobón et al. (Trujillo et al., 2017) conduct research on excombatants of the former Colombian armed conflict, who present a psychosocial and demographic profile very similar to that of the adolescent offenders (AOs) in the present study (Instituto Colombiano de Bienestar Familiar-ICBF, 2015; Comisión de la Verdad Colombia, 2019). The results point to atypical emotional processing in these individuals when presented with emotional stimuli drawn from the International Affective Picture System (IAPS). In this group, impaired social and cognitive abilities may be related to a poor empathic disposition (Papp et al., 2016), which, thus, establishes a link between emotional processing and empathy in actors of the armed conflict. Research suggests that AOs also report difficulty in recognizing facial emotions, such as disgust and anger, and in empathy in tasks involving real-life scenarios (Tobón et al., 2015; Pino et al., 2019). Moreover, AOs exhibit difficulty in executive function tasks known to tax top-down controlled mechanisms (Baluch and Itti, 2011; Gonzalez-Gadea et al., 2014). Therefore, the literature suggests that a delay in the maturation of the frontal lobes, especially the prefrontal cortex, may account for executive function impairments and poor control and programming of behaviors in AOs (Baluch and Itti, 2011). Evidence accrued to date proposes that the mechanisms responsible for the processing of emotional information presented in the focus of attention (i.e., via endogenous) are impaired in AOs (Tobón et al., 2015; Pino et al., 2019). This type of selective attention, together with exogenous attention, prioritizes relevant information according to its location or characteristic (Gonzalez-Gadea et al., 2014). While exogenous attention is automatic and driven by external stimuli that by their characteristics or properties stand out and attract the subject’s visual field, endogenous attention is voluntary and goal-oriented where in the presence of a relevant stimulus, the subject at will directs his or her visual field toward that stimulus, focusing on it and ignoring other stimuli in the environment (Zhou and Liu, 2013; Katsuki and Constantinidis, 2014). These two types of attention may have different performances when processing information. For example, endogenous attention may be more affected by interference and demands more cognitive resources compared to exogenous attention which may affect performance in terms of processing speed (Nguyen et al., 2020). Against this background, we hypothesize that responses to stimuli lying in the focus of attention are atypical in AOs. Alternatively, whether or not the mechanisms responsible for allocating attention to events occurring in the periphery of attention (i.e., exogenous), which convey emotional information, are also altered in these young offenders remains unknown.

The main objective of this study was to investigate the latter aspect using the emotional flanker task designed by Parra et al. (2018), which assesses whether or not attention is diverted from the central target or image when it is surrounded by four flanking images that display emotional information. Using this version of the emotional flanker task, the study demonstrated that such a response bias can be observed not only in individuals anxiety disorders but also in neurotypical individuals. The authors observed this attention bias using supraliminal or subliminal stimulus presentation and regardless of the information competing for attention. Similarly, evidence that demonstrates that attention bias to threat can be an exogenously or endogenously generated response and that the attention mechanisms driving it are dependent on the psychophysical (i.e., valence, arousal, and illuminance; Tipples and Sharma, 1953; Fox and Damjanovic, 2006; Lakens et al., 2013) and temporal properties of sensory experiences has accrued (Trujillo et al., 2021). For instance, this version of the emotional flanker task was adapted to an event-related potential (ERP) setting. Trujillo et al. (2021) recently demonstrated that the neural mechanisms also involved in endogenous attention to emotion can underpin the proposed exogenous component of this response, that is, the emotional bias to threat (Parra et al., 2018), when the encoding time is sufficiently long. The authors suggested that exogenous and endogenous attention mechanisms will be available to ensure the elicitation of such an adaptive behavior.

To provide a better theoretical context for the current study, recalling that the emotional flanker task was originally formulated with consideration of the biased competition model of attention by Desimone and Duncan (1995) is necessary (Yiend, 2010). This model proposes that when stimuli compete for attention, bias can be driven by bottom-up (exogenous) or top-down (endogenous) attention. These attention mechanisms can coexist and compete (Petersen and Posner, 2012). Indeed, when the environment exposes individuals to potentially threatening situations, central information processing systems are required to prioritize the aspects of the stimulus relevant to performing actions that ensure survival (Harrison et al., 2015). Thus, attention represents the gateway through which such prioritization is granted, and converging evidence confirms that threating stimuli receive such a priority (i.e., they bias attention) whether or not they lie in the focus or periphery of attention (Yiend and Mathews, 2005; Horstmann et al., 2006; Frewen et al., 2008; Yiend, 2010; Carretié, 2014; Parra et al., 2018; Trujillo et al., 2021). In fact, evidence suggests that threat-related information can be processed automatically when presented exogenously, which reinforces the notion of the adaptive value of such behaviors (Tipples and Sharma, 1953; Desimone and Duncan, 1995; Yiend and Mathews, 2005; Calvo et al., 2006; Fox and Damjanovic, 2006; Horstmann et al., 2006; Frewen et al., 2008; Yiend, 2010; Petersen and Posner, 2012; Lakens et al., 2013; Zhou and Liu, 2013; Carretié, 2014; Katsuki and Constantinidis, 2014; Harrison et al., 2015; Pool et al., 2016; Parra et al., 2018; Nguyen et al., 2020; Trujillo et al., 2021).

The threat superiority effect is the outcome of such a competition, which denotes the prioritization that the attention system grants to dangerous over nondangerous stimuli regardless of the nature of such stimuli (i.e., natural or man-made). This aspect includes whether or not they fall within or outside the focus of attention (Hansen and Hansen, 1988; Fox et al., 2007; Torralva et al., 2009; Subra et al., 2018). Moreover, Yiend and Mathews (2005) proposed that an important implication of biased competition models is that evidence of selective attention to emotions is only observed when the conditions of stimulus presentation enable competition. The author suggested that tasks that separately present emotional valances (e.g., threat versus neutral) and compare between them do not provide evidence of selective attention to either valances. This notion was in contrast with tasks that simultaneously present two stimuli and, therefore, in direct competition, does. To the best of our knowledge, the available literature on emotional processing, social cognition, and AOs has not considered the biased competition models of attention. This aspect is important, because they present compromised exogenous attention mechanisms in addition to atypical endogenous responses to emotion-laden stimuli, as previously reported in AOs (Gonzalez-Gadea et al., 2014). This result can pose significant implications for the understanding of their behaviors as well as for intervening with such behaviors.

## 2 Materials and methods

### 2.1 Participants

This study used nonprobabilistic sampling to recruit 39 male AOs aged between 14 and 18 years. The participants were residents of a reform center for young male offenders in Barranquilla, Colombia, where they fulfilled sentences for various punishable crimes such as sexual abuse, intentional homicide, aggravated crimes against freedom, integrity, theft, and drug possession. Furthermore, the study recruited 39 nonoffender adolescents from schools in the same city as control. The inclusion criteria for this group were ages ranging from 14 to 18 years, being male, level of education of less than 15 years of education, and not having committed a crime. Both groups were free from psychiatric or neurological diseases and were not under pharmacological treatment during the assessment. Although the majority of AOs displayed histories of drug and/or alcohol use, none were diagnosed with addiction or treated for this reason. They were given an informational form that describes the study and asked to sign an assent along with parental consent. The Ethics Committee of the Autonomous University of the Caribbean reviewed and approved the study.

### 2.2 Assessments

#### 2.2.1 Cognitive measures

To obtain information on cognitive functioning, we used the INECO (Instituto de Neurología Cognitiva) Frontal Screening (IFS), a brief and validated instrument (Torralva et al., 2009) that investigates executive function through subtests such as motor programming, verbal inhibitory control, conflicting instructions, GO/NO GO, spatial working memory, digit repetition and abstraction ability (interpretation of proverbs). It exhibits a specificity of 0.91 and a sensitivity of 0.96. The maximum score for the IFS is 30 points.

We also used the Montreal Cognitive Assessment (MOCA), which is a cognitive screening test used for detecting cognitive impairment. This instrument evaluates executive function, visuospatial processing, language skills, orientation, memory, abstraction, and attention. Although MOCA is a screening tool developed for assessing global cognitive abilities in older adults at risk of dementia (Carson et al., 2018), it provides detailed information on certain cognitive domains that can help identify neurocognitive profiles in neuropsychiatric disorders (D’Hondt et al., 2018).

In the absence of normative data for the adolescent population, the cut-off point taken as a reference was 28 (range: 22–30; *p* < 0.001). Pike et al. (2017) established this value for a sample of healthy adolescent controls that produced a sensitivity of 0.75 and a specificity of 0.90.

#### 2.2.2 Emotional processing

We evaluated emotional processing using two computational tasks conducted in E-Prime (Psychology Software Tools Inc, 1996).

#### 2.2.3 Emotional flanker task

**Figure 1:**
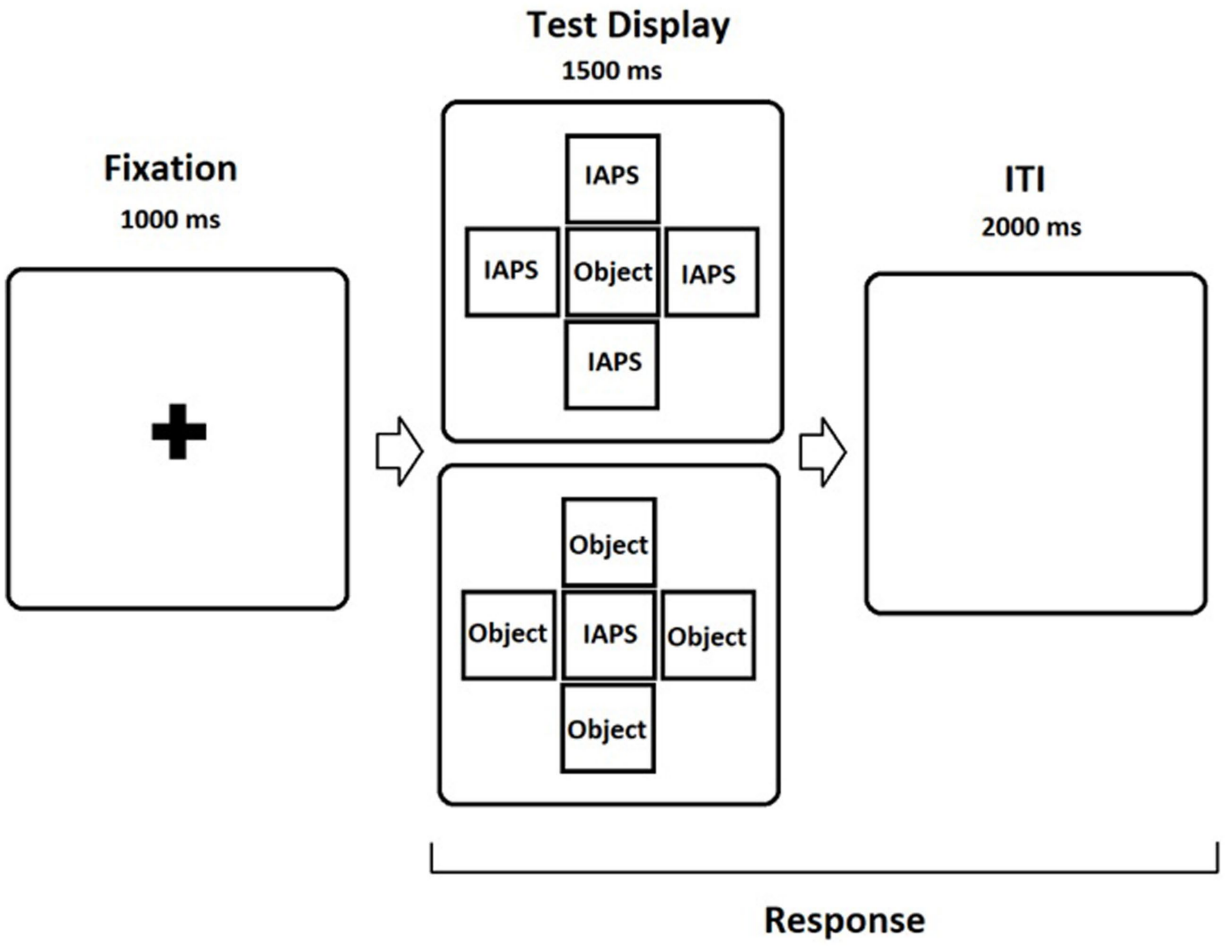
Trial sequence for the emotional flanker task. IAPS images could be either threatening or neutral and objects could be either living or non-living (see text for more details). When presented as flankers, the same IAPS image or the same object appeared in the four positions. Participants always responded to the target and ignored the flankers.

Parra et al. (2018) developed this task (Experiment 3), which was recently used by Trujillo et al. (2021) in an ERP study. The task presents four conditions, which are defined according to the content and position of emotionally relevant stimuli. It uses emotional images from the IAPS and line drawings (i.e., objects; Szekely et al., 2004) to promote competition for attention and to avoid stimuli congruency effects (i.e., the task excludes trials in which Targets and Flankers are the same type of stimulus). The study selected 60 threatening images and 60 neutral images from the IAPS normative database using valence and arousal as suggested by Lang et al. (1999; *threatening stimuli*, valence, M = 2.7, SD = 0.7, arousal, M = 6.0, SD = 0.8, *neutral stimuli*: valence: M = 5.9, SD = 1.1; arousal: M = 3.4, SD = 0.9). According to normative data, the valence scores for negative images are low (e.g., <4) but increase for neutral and positive images. In addition to the IAPS images, the study selected 30 living (e.g., cat) and 30 nonliving (e.g., broom) objects from the International Picture Naming Project database (Szekely et al., 2004) with naming frequencies >80%. The conditions of the tasks are target neutral/flanker object, target object/flanker neutral, target threat/flanker object, and target object/flanker threat (Figure 1). Trials belonging under each condition were randomized and divided in four blocks. Specifically, each block consisted of 60 trials using the layout presented in Figure 1 (240 trials in total). The duration of the task is approximately 20 min. Trials begin with a fixation cross presented for 1,000 ms followed by a test display presented for 1,500 ms. The participants were requested to press one of two previously allocated keys of a standard keyboard as quickly and accurately as possible dependent on whether or not a nonliving object or threatening image (key “v”) or a living object or neutral image (key “n”) appeared as the target (central image). They were instructed to ignore images presented as flankers (the same image shown in the four peripheral locations). This sequence was followed by an interval of 2,000 ms during which responses were still recorded (Figure 1). We measured accuracy and response time (RT) for the four conditions.

#### 2.2.4 Emotional screening task

This task was used to determine that the perceived emotions matched those reported by Lang et al. (1999). After completing the emotional flanker task, the participants completed this brief emotional screening test, which presented the same IAPS images shown in the emotional flanker task one at a time. They rated their perception of the images (i.e., neutral or violent) using a five-point Likert-type scale (1 = *Not at all*, 5 = Very much).

### 2.3 Procedures

Both groups, as well as their parents, signed informed assent following the guidelines of the Helsinki Declaration. The application of the different instruments were conducted at the reform center for AOs and completed at schools for nonoffender adolescents. Agreement was reached on the schedule with the directors of both centers to avoid interrupting various academic and recreational activities. The participants first performed the emotional flanker task followed by the emotional screening task. Both tasks were performed in one session, and the INECO and MOCA were completed in a second session to avoid fatigue. An important aspect to mention is that, as the members of each group were selected, the following sociodemographic data were recorded: age, years of education, and socioeconomic status. For the AOs sample, these data were obtained from admission and follow-up records from the center in which they are institutionalized. Information corresponding to the control group was derived from the demographic data section of the informed consent form.

### 2.4 Data analysis

The study used the Statistical Package for the Social Sciences version 26.0. Sociodemographic data, IFS, MOCA, and responses to the emotional screening test were compared between groups using Student’s *t*-test for independent means. Data from the emotional flanker task were analyzed using a general linear mixed model (ANOVA) that included two within-subject (factors and emotion; neutral and threat) and position (central and peripheral). The between-subject factor was group (control and offenders). This scheme led to a 2 × 2 × 2 design comprising four repeated measures (i.e., target neutral/flanker object, target object/flanker neutral, target threat/flanker object, and target object/flanker threat) and the two groups. We ran two mixed models: one for accuracy, and the other for RT. The significance threshold was set to alpha = 0.05 for all analyses.

## 3 Results

Table 1 presents the demographic and background cognitive measures. Significant differences between groups were observed for age [*t*(65,1) = 4,355, *p* < 0.05], socioeconomic level [*t*(62,0) = −13,091, *p* < 0.05], and level of education [years; *t*(76) = −3,422, *p* < 0.05]. The IFS demonstrated that AOs exhibited significantly lower performance on motor programming [*t*(46,7) = 2,908, *p* < 0.05], verbal inhibitory control [*t*(67,4) = 3,567, *p* < 0.05], numerical working memory [*t*(63,7) = 6,11, *p* < 0.05], visual working memory [*t*(76) = 7,345, *p* < 0.05], and the global score for IFS [*t*(60,2) = 7,476, *p* < 0.05]. We also found significant differences in MOCA, specifically in visuospatial/executive function [*t*(76) = 2,387, *p* < 0.05], identification [*t*(45,5) = 2,593, *p* < 0.05], attention [*t*(70,6) = 2,008, *p* < 0,05], abstraction [*t*(54,6) = 5,114, *p* < 0.05], and the global score for MOCA [*t*(65,0) = 4,633, *p* < 0.05].

**Table 1:** Demographic and cognitive data for adolescent offenders and non-offenders and outcomes from between-group comparisons.

	Adolescent offenders (N = 39)		Adolescent non-offenders (N = 39)		p*

	M	SD	M	SD	
Age	17.31	0.800	16.28	1.23	0.000
Socioeconomic status	1.08	0.270	2.18	0.451	0.000
Education (years)	8.67	2.14	10.23	1.88	0.001
IFS					
Motor programming	2.384	0.989	2.871	0.338	0.006*
Interference resistance	2.897	0.307	2.846	0.431	0.547
Inhibitory motor control	2.846	0.539	2.769	0.426	0.487
Verbal inhibitory control	3.282	1.84	4.564	1.27	0.001*
Verbal working memory	1.205	0.893	1.487	0.643	0.114
Numerical working memory	1.153	0.932	2.230	0.583	0.000*
Visual working memory	1.743	1.01	3.256	0.785	0.000*
Abstraction capacity	1.794	0.614	2.012	0.692	0.146
IFS global score	17.307	3.43	22.038	1.95	0.000*
MOCA					
Visuospatial/executive	4.000	1.05	4.487	0.720	0.019*
Identification	2.641	0.706	2.948	0.223	0.013*
Memory deferred recall	3.564	1.33	3.666	1.08	0.710
Attention	4.461	1.27	4.974	0.959	0.048
Language	2.461	0.682	2.487	0.601	0.861
Abstraction	1.230	0.705	1.871	0.338	0.000*
Orientation	5.897	0.307	5.974	0.160	0.171
MOCA global score	24.92	2.75	27.35	1.78	0.000*

*Analyses that yielded significant group differences after matching groups for years of education [39 adolescent offenders vs. 39 adolescent non-offenders; *t*(62.74) = 1.74, *p* = 0.09].

### 3.1 Emotional flanker task

#### 3.1.1 Accuracy

**Figure 2:**
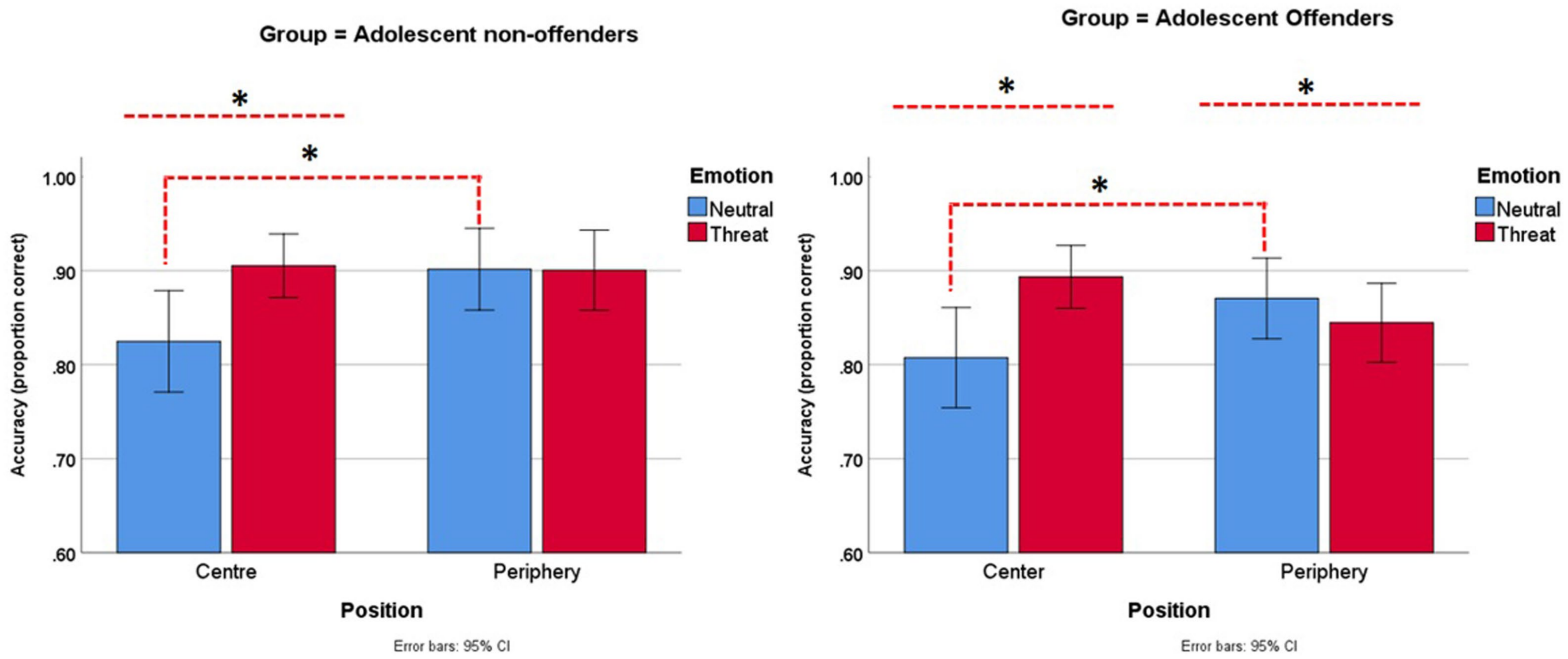
Mean accuracy data by group across the four conditions of the emotional flanker task (*reached the significance threshold, see Supplementary Table 2 for means and standard deviations).

The mixed ANOVA model revealed a nonsignificant main effect of group [*F*(1,77) = 1.43, *p* = 0.235, η^2^ = 0.02]. The effect of emotion was significant [*F*(1,77) = 14.47, *p* < 0.001, η^2^ = 0.16] but not position [*F*(1,77) = 2.39, *p* = 0.126, η^2^ = 0.03]. Relevant to the hypothesis are the emotion × position and three-way emotion × position × group interactions. The former proved significant [*F*(1,77) = 22.89, *p* < 0.001, η^2^ = 0.23] but not the latter [*F*(1,77) = 0.56, *p* = 0.457, η^2^ = 0.01]. Furthermore, the study conducted Bonferroni’s corrected paired-sample *t*-tests (alpha = 0.0125) to explore the emotion × position interaction. The results confirmed that threatening images were associated with higher accuracy compared with neutral images when presented as targets [*t*(78) = 4.05, *p* < 0.001]. Moreover, Neutral images led to lower accuracy when presented as targets than when they appeared as flankers [*t*(78) = 3.47, *p* = 0.001]. Threatening images presented as flankers led to lower accuracy than did neutral images presented as flankers. This effect that was seemingly driven by AOs and indicated significance [*t*(78) = 2.57, *p* = 0.012]. No other contrast reached the adjusted threshold. Figure 2 presents the interaction graphs.

#### 3.1.2 Response time

**Figure 3:**
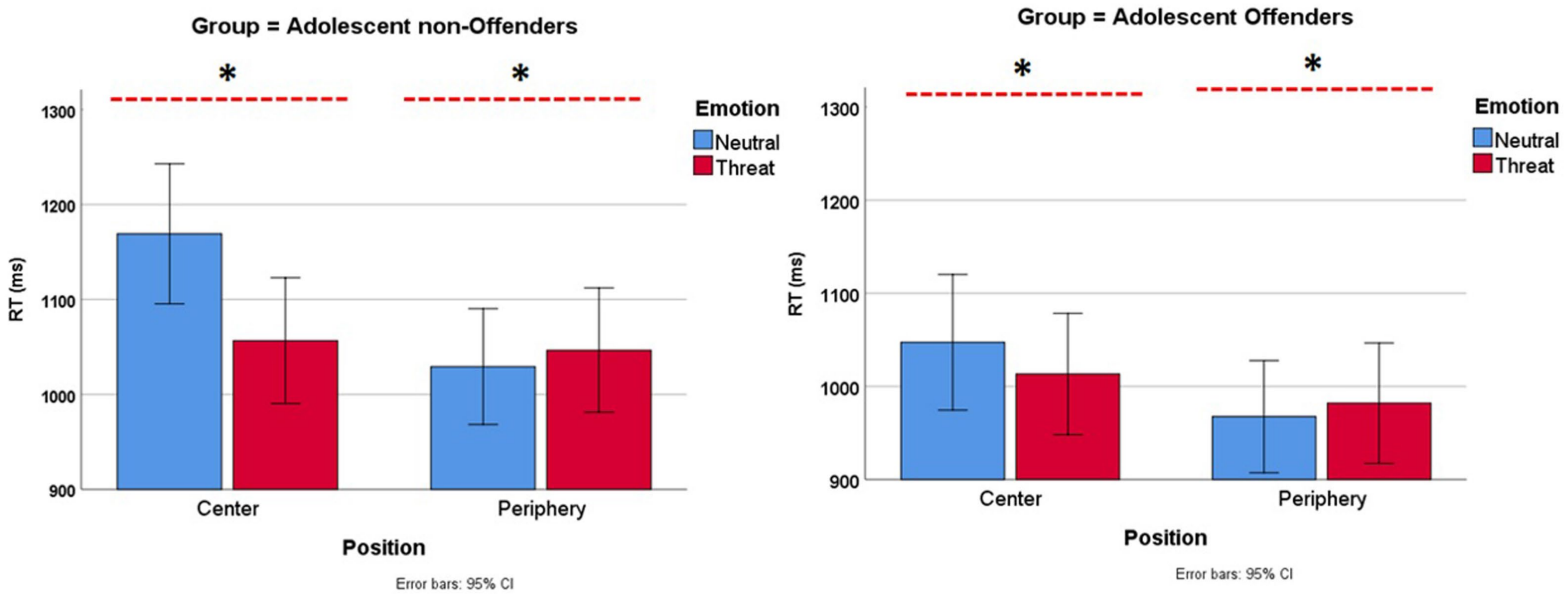
Mean reaction time data by group across the four conditions of the emotional flanker task (*reached the significance threshold, see Supplementary Table 2 for means and standard deviations).

The mixed ANOVA model revealed a nonsignificant main effect of group [*F*(1,77) = 2.66, *p* = 0.107, η^2^ = 0.03], and the effect of emotion was significant [*F*(1,77) = 10.62, *p* = 0.002, η^2^ = 0.12] as well as position [*F*(1,77) = 59.12, *p* < 0.001, η^2^ = 0.43]. Both interactions were significant: emotion × position [threat-related bias; *F*(1,77) = 26.29, *p* < 0.001, η^2^ = 0.25] and the three-way emotion × position × group [*F*(1,77) = 5.44, *p* = 0.022, η^2^ = 0.07]. Furthermore, we performed Bonferroni’s corrected paired-sample *t*-tests to follow up on the former interaction (alpha = 0.0125) and confirmed that threatening images presented as targets were detected faster than were neutral images [*t*(78) = 4.45, *p* < 0.001]. However, threatening images presented as flankers decreased RTs to targets compared with neutral images; this effect was deemed significant [*t*(78) = 2.15, *p* = 0.035]. Neutral images as targets were detected more slowly [*t*(78) = 7.94, *p* < 0.001] but did not influence performance when they appeared as flankers. To explore the three-way interaction, data on RTs were collapsed across emotions for each position (threat-related bias = RT threat periphery − RT neutral periphery; target effect = RT threat center − RT neutral center). Moreover, the flanker and target effects were compared across groups using independent-sample t-tests. The result revealed significant differences in the target [*t*(63.35) = 2.46, *p* = 0.017] but not in the flankers [*t*(61.01) = 0.18, *p* = 0.854]. No other contrast reached the adjusted threshold. Figure 3 presents the interaction graphs.

Correlation analysis involving RT and accuracy confirmed that the reported effects are not result of a trade-off between speed and accuracy (see Supplementary Table 1 for the correlation table). Furthermore, to explore whether or not differences in education between groups accounted for the three-way interaction, we reran the model, including the matched subsamples described in the analysis of the cognitive background data (see footnote for Table 1). All previously reported effects remained significant when these education-matched groups were included in the mixed ANOVA.

To assess if potential differences in perception of emotional valence could explain the reported effects, we compared the valence ratings for the emotional screening task between the groups (Table 2). The perceived valances closely matched those selected from the IAPS database. Moreover, such ratings did not significantly differ across groups, which suggests that differences in the perception of emotional valance would unlikely explain the effects observed during the emotional flanker task.

**Table 2:** Ratings of the emotional screening task.

		Adolescent offenders (*N* = 39)		Adolescent non-offenders (*N* = 39)		
Presented valance	Rated valence	M	SD	M	SD	p*
Neutral	Neutral	4.69	0.553	4.67	0.669	0.883
Neutral	Threat	1.36	0.706	1.33	0.649	0.867
Threat	Neutral	1.48	0.610	1.42	0.880	0.719
Threat	Threat	4.45	0.514	4.59	0.686	0.324

Finally, to investigate whether or not general cognitive abilities (as assessed using the MOCA) or frontal lobe functions (as assessed by the IFS) could account for the significant three-way interaction in RT (i.e., data linked to the emotional bias to threat), we ran 2 three-way ANCOVA models that controlled for the effects of such functions (i.e., entering total MOCA and total IFS as covariates). Controlling for the influence of general cognitive functions (MOCA) did not eliminate the three-way interaction [*F*(1,77) = 9.34, *p* = 0.003, η^2^ = 0.12], whereas controlling for frontal lobe functions did [*F*(1,77) = 2.83, *p* < 0.097, η^2^ = 0.036]. Notably, such a controlled analysis also omitted the key emotion × position interaction, which implies an influence beyond the AO group.

## 4 Discussion

The present study aimed to investigate whether or not the mechanisms responsible for allocating attention to endogenous and exogenous stimuli that convey emotional information are altered in AOs. We also investigated the underpinnings of atypical responses to such a process. Analyses revealed several key findings. First, AOs presented atypical general cognitive and frontal lobe functions that were not explained by level of education. Second, while the influence of threatening flankers on responses to targets revealed a normal threat-related bias in this group (which proposes preserved exogenous attention to emotional stimuli), their responses to emotional targets differed from those observed in nonoffenders. Such atypical responses indicated an enhanced reactivity to and faster discrimination of emotional information, which is an effect for which level of education could not account. Third, these atypical responses in AOs were unlikely to have been driven by the atypical perceptual discrimination of emotions or general cognitive abilities (i.e., MOCA). However, the findings implied that frontal lobe functions (IFS) could account for this result.

### 4.1 Atypical cognitive and frontal lobe functions in adolescent offenders

In the present sample, AOs produced lower scores on MOCA compared with nonoffenders (although the scores in general were high and unlikely to indicate significant cognitive impairment). Notably, such differences persisted after omitting group differences in education. A specific assessment of frontal lobe functions using the IFS revealed that AOs presented with executive function impairments. Scores below 25 were reported as suitable for the detection of executive dysfunction in patients who present alterations related to the frontostriatal circuits; scores equal or close to 30 indicate preserved executive functions (Torralva et al., 2009). Gonzalez-Gadea et al. (2014) previously reported executive function impairments in AOs from similar populations (for a meta-analysis, see Gil-Fenoy et al., 2018 and Jiang et al., 2020). Such cognitive disorders share biological underpinnings with the behavioral problems displayed by these young individuals in which abnormalities in the maturation and expression of the frontal lobe seem to account for both (Borrani et al., 2015; Borrás et al., 2017). A potential explanation for these results is that chronic exposure to negative environments (e.g., violent, drug use, and childhood abuse) may impede the normal developmental trajectories of frontal lobe functions, which leads to maladaptive behaviors. For instance, previous studies demonstrated that institutionalized adolescents raised under social deprivation conditions displayed delayed development in relation to theory of mind (ToM) relative to adolescents raised with their biological families (Baez et al., 2018). This finding implied that the development of moral cognition is vulnerable to the impact of institutionalization. Taken together, previous studies and the current results reveal that the discrepancies observed between the groups may be associated with potential abnormalities in the frontal lobe. However, further research is required to identify whether or not these anomalies are a result of inadequate parenting and/or adverse rearing conditions, which would then increase the risks of maladaptive behaviors.

### 4.2 Attention bias to threat in adolescent offenders

Based on previous studies (Baez et al., 2018), we expected that AOs would be less capable of identifying emotional valences from IAPS images than would adolescent nonoffenders. However, this prediction was not the case. Not only were AOs equally accurate compared with nonoffenders in detecting emotional valence, but they were also faster. A possible explanation for such a departure from our predictions remains unclear. One possibility is the task themselves. Other studies (Gonzalez-Gadea et al., 2014) that reported impaired social cognition (emotion recognition and ToM) in AOs used tasks in which participants conduct an emotional assessment of presented stimuli (e.g., positive, negative, angry, and happy). The emotional flanker task requires participants to decide if the IAPS images presented are violent (i.e., threatening condition) or neutral. Deciding whether or not an event is violent or if it conveys a particular type of emotion may rely on different underlying mechanisms.

Evidence suggests that offenders regardless of age display attentional bias to violence-related and negative stimuli (Mellentin et al., 2015). Psychopathic traits only seemingly, partially explain such a bias; however, negative childhood experiences appears to account for it. Domes et al. (2013) cited that enhanced attention to violence-related stimuli in criminal offenders is associated with adverse developmental experiences and delinquency. Moreover, they put forward that this is carried into adulthood (Domes et al., 2013; Mellentin et al., 2015). Kimonis et al. (2008) observed that youth with high callous–unemotional traits and enhanced orientation toward distressing stimuli exhibited stronger histories of abuse. This result reinforced the notion that environmental factors seemingly influence the development of these traits. Such a bias appears to be very pronounced that even when presented with ambiguous situations, boys with conduct problems tend to interpret these stimuli as hostile regardless of levels of callous–unemotional traits (Hartmann et al., 2020). The latter author suggests that the interaction of attentional and attributional biases in children with conduct problems may contribute to increased aggressive behaviors. With respect to the previous point and in view of future research, exploring the attentional bias of AOs according to low or high levels of callous–unemotional traits is important. The reason is that they, as well as young people with behavioral problems, will be a very heterogeneous group and, therefore, would present diverse patterns of behavioral (reactive or proactive aggression) and neural responses to affective stimuli (Viding et al., 2012).

Finally, findings are in agreement with the literature. Classical attention bias to threat was evident not only in relation to RT (which is the variable revealing these effects) but also in the measurement of accuracy in target detection. Figure 2 depicts that AOs, relative to nonoffenders, were less accurate in identifying object targets when these were flanked by threating compared with neutral stimuli. This effect approached Bonferroni’s corrected threshold (*p* = 0.012). None of previous studies using the emotional flanker task has observed such an attention bias to threat in relation to accuracy (Tipples and Sharma, 1953; Fox and Damjanovic, 2006; Lakens et al., 2013; Parra et al., 2018; Trujillo et al., 2021). RT is known to be a more sensitive variable for revealing the subtle effects of emotion processing (normal or altered) on attention (Yiend and Mathews, 2005; Yiend, 2010; Petersen and Posner, 2012; Harrison et al., 2015). Such a reduced inhibition of task-irrelevant emotional information has been previously reported in batteries using an emotional Stroop task (Chan et al., 2010).

One may argued that the long-term exposure employed in the task should have enabled the endogenous processing of flankers. However, Parra et al. (2018) and Trujillo et al. (2021) previously illustrated that emotional bias to threat is observed when exposure time ranges from subliminal (200 ms) to supraliminal (>1,000 ms). This finding has led to the proposal of the adaptive nature of this response, which seemingly plays a key role in survival. The results presented further support for this notion, as if longer exposure time would have encouraged the endogenous processing of flanker images. Moreover, the current study should have observed changes in AOs similar to those observed for response-relevant (targets) threat-images. However, the opposite was true. On the contrary, threat-related bias remained uninfluenced. Given the previous studies and the evidence presented, the researcher feel confident in suggesting that the processing of endogenous stimuli of flankers should be omitted as drivers of the reported effects.

To the best of our knowledge, this study is the first to report on the presence of attention bias to threat for endogenous and exogenous stimuli in AOs. This finding calls for additional research on attention in these young people and raises further questions. For instance, are the perceptions of violence as a dichotomous decision and emotional valences dissociable in offenders? Which type of information would carry more value in mitigating the impact of chronic exposure to negative experiences via social cognition interventions? Could improving emotion recognition reduce the perception and perpetration of violence in such individuals (Tipples and Sharma, 1953; Hansen and Hansen, 1988; Desimone and Duncan, 1995; Psychology Software Tools Inc, 1996; Lang et al., 1999; Szekely et al., 2004; Yiend and Mathews, 2005; Calvo et al., 2006; Fox and Damjanovic, 2006; Horstmann et al., 2006; Fox et al., 2007; Frewen et al., 2008; Kimonis et al., 2008; Torralva et al., 2009; Chan et al., 2010; Yiend, 2010; Baluch and Itti, 2011; Petersen and Posner, 2012; Viding et al., 2012; Domes et al., 2013; Lakens et al., 2013; Zhou and Liu, 2013; Bowen et al., 2014; Carretié, 2014; Gonzalez-Gadea et al., 2014; Katsuki and Constantinidis, 2014; Borrani et al., 2015; Harrison et al., 2015; Mellentin et al., 2015; Tobón et al., 2015; Pool et al., 2016; Borrás et al., 2017; Pike et al., 2017; Trujillo et al., 2017; Baez et al., 2018; Carson et al., 2018; D’Hondt et al., 2018; Gil-Fenoy et al., 2018; Parra et al., 2018; Subra et al., 2018; Pino et al., 2019; Hartmann et al., 2020; Jiang et al., 2020; Nguyen et al., 2020; Trujillo et al., 2021)?

### 4.3 Underpinnings of the attention bias to threat in adolescent offenders

We did not observe differences between AOs and nonoffenders in the ratings of the IAPS images after the emotional flanker task. As previously described, the participants rated the images in the task as violent or neutral using a Likert-type scale. Future studies could use a similar task to compare ratings on emotional valences (e.g., negative, unpleasant, and excited) and investigate whether or not they differ from violence/threatening ratings. This aspect is important, because we identified that low levels of education or general cognitive abilities (i.e., MOCA) do not account for such heightened responses in AOs (faster responses to IAPS images that reveal the target and flanker effects with the latter also appearing for accuracy). However, frontal lobe functions (i.e., executive functions as assessed by the IFS) seemingly accounted for these results. This study is not the first to report poor executive functions in AOs. Gonzalez-Gadea et al. (2014) also observed poor executive functions in AOs. However, their study did not observe associations between impairments and difficulties with emotion recognition and cognitive empathy.

The fact that we found such an association reinforces the notion that emotion, ToM, and perception of violence may be reliant on different neurocognitive mechanisms. Where the former two could more heavily rely on the anatomo-functional integrity of limbic structures, the latter may be more reliant on the influence of the cortical components of attention–action networks (Bick and Nelson, 2016). Recently, using ERP analysis, Trujillo et al. (2021) found that the target effect reported with the emotion flanker task is largely driven by the frontocentral components of the attention network involved in top-down regulation. Alternatively, threat-related bias is regulated by earlier centroparietal activity that supports top-down or bottom-up attention mechanisms according to task demands (Petersen and Posner, 2012). The findings presented demonstrated the need for further research to better characterize the biopsychosocial drivers of conduct disorders in male AOs. Such knowledge will exert a significant impact on intervention programs designed to prevent or reverse maladaptive behaviors in children and adolescent at risk.

### 4.4 Limitations and future directions

This study has its limitations. First, we worked with a modest sample size of 100% male subjects. However, the number of participants in the study was similar to that reported in previous research (Tipples and Sharma, 1953; Hansen and Hansen, 1988; Desimone and Duncan, 1995; Psychology Software Tools Inc, 1996; Lang et al., 1999; Szekely et al., 2004; Yiend and Mathews, 2005; Calvo et al., 2006; Fox and Damjanovic, 2006; Horstmann et al., 2006; Fox et al., 2007; Frewen et al., 2008; Torralva et al., 2009; Yiend, 2010; Baluch and Itti, 2011; Petersen and Posner, 2012; Domes et al., 2013; Lakens et al., 2013; Zhou and Liu, 2013; Carretié, 2014; Gonzalez-Gadea et al., 2014; Katsuki and Constantinidis, 2014; Borrani et al., 2015; Harrison et al., 2015; Mellentin et al., 2015; Pool et al., 2016; Borrás et al., 2017; Pike et al., 2017; Baez et al., 2018; Carson et al., 2018; D’Hondt et al., 2018; Gil-Fenoy et al., 2018; Parra et al., 2018; Subra et al., 2018; Jiang et al., 2020; Nguyen et al., 2020; Trujillo et al., 2021) on AOs. Thus, obtaining a larger sample that includes the female population would be pertinent. Second, the groups exhibited significant differences in age, level of education, and socioeconomic status, which were not controlled for during analyses for two reasons. First, data from the emotional flanker task did not comply with ANCOVA assumptions that in as much such covariates were strongly correlated with the grouping variable and only mildly correlated with the dependent variables. Nevertheless, such departures are interesting as Volavka noted in a model (Volavka, 1999). Education and socioeconomic status (e.g., rearing environment) are risk factors for antisocial or violent behaviors. Therefore, eliminating the variance accounted for by such factors can indeed omit the variance of interest. To avoid such methodological shortcomings, actions should be taken during recruitment in which groups are matched as close as possible according to these variables, moreover, samples should be sufficiently large to enable the categorization of demographic variables. Future studies should take the case of these suggestions to explore the extent to which such factors could be mediators of the effects reported here. Third, the present study used only psychometric measures and systematized activities to examine attentional bias. In the future, using neurophysiological and neuroimaging recording techniques will help researchers gain a better understanding of the modulations and neurobiological underpinnings of these cognitive and emotional information processing tasks in the reference population. Furthermore, extensive sociodemographic data should be considered, because the reported effects could be mediated by personal (e.g., educational attainment) and familiar (e.g., rearing environment) factors (Volavka, 1999). The reported data should also be considered together with affective status and individual dispositions (e.g., motivational, trait-anxiety, and negative emotions), which are known to be common comorbidities of these adolescents and to affect social cognition (Ryan and Redding, 2004).

## 5 Conclusion

The AOs presented with elevated responses to stimuli that convey violent information that appeared in the focus or periphery of attention. Low levels of education, poor discrimination of violent/neutral images, or poor general cognitive abilities did not account for such findings. However, poor frontal lobe functions seemingly accounted for them, which suggests that attentional bias to violent stimuli, which were previously reported for these young individuals, could be linked to underdeveloped frontal lobe functions. Future studies are required to examine the distinction between emotion and violence perception in AOs and their neurocognitive underpinnings.

## Data availability statement

The raw data supporting the conclusions of this article will be made available by the authors, without undue reservation. Requesting it to the correspondence author.

## Ethics statement

The studies involving humans were approved by Ethics Committee of the Autonomous University of the Caribbean. The studies were conducted in accordance with the local legislation and institutional requirements. Written informed consent for participation in this study was provided by the participants’ legal guardians/next of kin.

## Author contributions

MP, RR, GG, and MAP designed and conceived the study. GG, VP, and MAP performed the research. MP, RR, MAP, GG, and VP performed the data analysis. MP, RR, VP, and MAP: writing– review & manuscript edition. All authors contributed to the article and approved the submitted version.
