# Supplementary material for: Assessing attentional bias to emotions in adolescent offenders and nonoffenders
Source: Front Psychol. 2023 Nov 24;14:1192114. doi: 10.3389/fpsyg.2023.1192114 (PMC10704598; doi:10.3389/fpsyg.2023.1192114)
Supplement: Supplementary file 1 [file Table_1.pdf]

Supplementary Material

Table 1. Correlation analysis carried out to explore a potential speed/accuracy trade-off as the driver of effects reported in the manuscript.

REACTION TIME		ACCURACY: NEUTRAL CENTER	ACCURACY: NEUTRAL PERIPHERY	ACCURACY: THREAT CENTER	ACCURACY: THREAT PERIPHERY
**NEUTRAL CENTER**	**r**	-0.130	-0.056	-0.112	-0.037
	**p-value**	0.255	0.627	0.328	0.748
**NEUTRAL PERIPHERY**	**r**	-0.114	-0.220	-0.222	-0.195
	**p-value**	0.322	0.053	0.050	0.087
**THREAT CENTER**	**r**	-0.071	-0.075	**-.261***	-0.055
	**p-value**	0.537	0.514	**0.021**	0.631
**THREAT PERIPHERY**	**r**	-0.088	-0.187	-0.212	-0.175
	**p-value**	0.443	0.101	0.062	0.126

* Did not survived FDR correction (*q*-value = 0.248) (Pike, 2011)

References

Pike, N. (2011). Using false discovery rates for multiple comparisons in ecology and evolution. Methods in Ecology and Evolution, 2, 278-282. https://doi.org/10.1111/j.2041-210X.2010.00061.x
