# Supplementary material for: Assessing attentional bias to emotions in adolescent offenders and nonoffenders
Source: Front Psychol. 2023 Nov 24;14:1192114. doi: 10.3389/fpsyg.2023.1192114 (PMC10704598; doi:10.3389/fpsyg.2023.1192114)
Supplement: Supplementary file 2 [file Table_2.pdf]

Supplementary Material

Table 2. Descriptive Statistics for the variables of the Emotional Flanker task across groups.

		Reaction Time		Accuracy	
	**Group**	**Mean**	**SD**	**Mean**	**SD**
Controls	Target Neutral	1169.14	282.19	0.82	0.16
	Flanker Neutral	1029.26	212.82	0.90	0.09
	Target Threat	1056.63	229.71	0.91	0.08
	Flanker Threat	1046.59	233.85	0.90	0.08
Adolescent Offenders	Target Neutral	1049.25	169.35	0.81	0.18
	Flanker Neutral	965.29	169.80	0.88	0.16
	Target Threat	1010.42	184.47	0.89	0.13
	Flanker Threat	980.34	175.33	0.85	0.16
