# Supplementary material for: Assessing attentional bias to emotions in adolescent offenders and nonoffenders
Source: Front Psychol. 2023 Nov 24;14:1192114. doi: 10.3389/fpsyg.2023.1192114 (PMC10704598; doi:10.3389/fpsyg.2023.1192114)
Supplement: Supplementary file 3 [file Table_3.pdf]

Supplementary Material

Table 3. Types of crimes committed by Adolescents Offenders (AOs)

**Types of crime**	**Adolescent Offenders (N = 39)**
Sexual Abuse	8 (20,5%)
Homicide	16 (41%)
Extortion	2 (5,1%)
Illegal possession of weapons	5 (12,8%)
Theft	6 (15,4%)
Receiving	1 (2,6%)
Illegal possession of narcotics	1 (2,6%)
